# Smoking status among cancer patients by specialty: A U.S. nationwide representative analysis

**DOI:** 10.1002/cam4.6684

**Published:** 2023-11-20

**Authors:** José Ignacio Nolazco, Yuzhe Tang, Khalid Y. Alkhatib, Andrew J. King, Matthew Mossanen, Steven Lee Chang

**Affiliations:** ^1^ Division of Urological Surgery Brigham and Women's Hospital, Harvard Medical School Boston Massachusetts USA; ^2^ Servicio de Urología, Hospital Universitario Austral, Universidad Austral Pilar Argentina; ^3^ Urology Department, Beijing Tsinghua Changgung Hospital School of Clinical Medicine Tsinghua University Beijing China; ^4^ Center for Surgery and Public Health, Brigham and Women's Hospital, Harvard Medical School Boston Massachusetts USA; ^5^ Division of Urology University of Pennsylvania Philadelphia Pennsylvania USA; ^6^ Department of Health Care Policy Harvard Medical School Boston Massachusetts USA; ^7^ Department of Radiation Oncology Brigham and Women's Hospital Boston Massachusetts USA; ^8^ Lank Center for Genitourinary Oncology, Dana‐Farber Cancer Institute Boston Massachusetts USA

**Keywords:** cancer survivors, physicians, smoking cessation, tobacco

## Abstract

**Background:**

Persistence in tobacco use among cancer survivors has been associated with a multitude of clinicodemographic factors. However, there is a paucity of understanding regarding the role the healthcare professional's specialty plays in tobacco cessation in tobacco‐related cancer survivors.

**Methods:**

We conducted a cross‐sectional analysis of data from cancer survivors with a smoking history using the Behavioral Risk Factor Surveillance System (BRFSS) database to examine differences in the proportion of patients continuing tobacco use among patients with a diagnosis of cancer segregated by cancer site specialty over the 2016–2020 period. We accounted for complex survey design and used sampling weights to obtain a nationwide representative sample. We employed modified Poisson regression adjusting for age, gender, education, income, race, marital status, and medical specialty.

**Results:**

We analyzed 19,855 cancer survivors with a current or past history of tobacco use, of whom 5222 (26,3%) self‐reported to be current smokers. Patients with urological and gynecological tobacco‐related malignancies had a higher relative risk (RR) of being current smokers with a RR of 1.30 (95% confidence interval, 1.12–1.51) and 1.25 (95% confidence interval, 1.12–1.39) respectively. Malignant Hematology had the lowest RR of smoking status among all other specialties RR 0.85 (95% confidence interval, 0.59–1.21).

**Conclusions:**

Continuing smoking rates among tobacco‐related cancer survivors were different between specialties. One in four cancer survivors were current smokers; this emphasizes health professionals' paramount role in tobacco cessation counseling.

## Graphical abstract

This study analyzed data from cancer survivors with a smoking history using the BRFSS database to examine differences in the proportion of patients continuing tobacco use among patients with a diagnosis of cancer segregated by cancer site specialty over the 2016–2020 period. Results showed that patients with urological and gynecological tobacco‐related malignancies had a higher relative risk of being current smokers, while malignant hematology had the lowest relative risk. The study concludes that continuing smoking rates among tobacco‐related cancer survivors differ between specialties and highlights the importance of health professionals in tobacco cessation counseling.

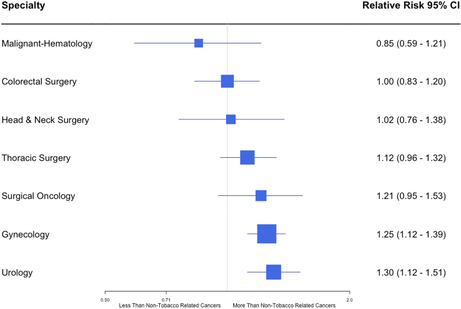

## 1 INTRODUCTION

Smoking is the leading preventable cause of death worldwide.^1^ In 2019, approximately one‐fifth of U.S. adults (50.6 million) reported being current users of some form of tobacco.^2^ Tobacco is associated with many types of cancer, including lung, laryngeal, oral, esophageal, pharyngeal, bladder, kidney, liver, gastric, pancreatic, colon rectum, cervical, and acute myeloid leukemia.^3^ Continued smoking among cancer patients can increase the odds ratio of disease recurrence and progression^4^, ^5^, ^6^ decrease the quality of life for cancer survivors^7^, ^8^ and places this population at an increased risk of developing second primary malignancies.^9^, ^10^

Because of the deleterious aspects of tobacco use, the diagnosis of cancer may serve as a “turning point” leading some patients to adopt a healthier lifestyle after a cancer diagnosis and quit smoking. At the same time, other patients in a similar situation fail to institute any meaningful change and will continue to smoke. Although prior studies have identified patient factors associated with tobacco cessation versus continued smoking among cancer patients, there remains an incomplete understanding of this multifactorial issue.

Although prior investigations have focused primarily on patient characteristics, it is recognized that medical counseling plays a fundamental role in tobacco cessation; therefore, the tobacco cessation rate might be substantially influenced by the healthcare professional factor.^11^ While some physicians actively defer a discussion regarding tobacco cessation to other healthcare professionals, other physicians may actively incorporate this discussion to capitalize on the “teachable moment” of cancer diagnosis to pursue this challenging task of tobacco cessation. It is critically important to identify all the risk factors associated with continuing to smoke in cancer patients to increase the effectiveness of tobacco cessation efforts. Some studies have investigated the factors associated with continuing to smoke after cancer diagnosis.^12^, ^13^, ^14^, ^15^, ^16^ However, no previous studies have evaluated the relationship between continuing smoking after cancer diagnosis and cancer site by the medical or surgical specialty which primarily manages the disease. It is unknown if certain specialties are associated with higher smoking rates in cancer patient survivors. We hypothesize that there is a difference in continuing smoking depending on cancer type by specialty. This study aimed to test the hypothesis that different medical specialties are associated with different tobacco cessation rates in tobacco‐related cancer patients.

## 2 MATERIALS AND METHODS

### 2.1 Study design/data source

We perform a cross‐sectional study using the BRFSS (Behavioral Risk Factor Surveillance System) database. This database is the largest continuously conducted health survey in the world, which collects data using telephone surveys of >400,000 adults each year from all 50 states as well as the District of Columbia and three U.S. territories. BRFSS is conducted by the Center for Disease Control and Prevention (CDC's) National Center for Chronic Disease Prevention and Health Promotion (NCCDPHP) and collects data about health‐related risk behaviors, chronic health conditions, and the use of preventive services.^17^

BRFSS collects comprehensive and detailed data about participants' health status. This data includes demographic (including age, education, income, and occupation), health status (including several chronic conditions such as hypertension, diabetes, and arthritis), health history (history of cancer, type of cancer, and course of treatment), healthcare access and behavioral data (exercise, tobacco use, marijuana use, alcohol consumption, diet, vaccination status) among others. We appended data from the years 2016 to 2020 BRFSS annual surveys assembling the analyzed dataset.

### 2.2 Study cohort

The study cohort was comprised of individuals aged 18 years or older who reported a history of cancer and a current or past smoking history. Cancer survivor patients were identified based on their responses to the survey question, “What type of cancer was it?” (Variable name: CNCRTYP1). Current and former smokers were identified through the survey question SMQ100 “Have you ever smoked more than 100 cigarettes in your life?”. Among individuals with a smoking history, smokers were classified as those who continue to smoke or quit smoking based on their response to SMOKDAY2 “Do you now smoke cigarettes every day, some days, or not at all?” (Table S1). We divided the cancer cohort into tobacco‐related cancers and nontobacco‐related cancers. We define tobacco‐related cancers as those specified on the National Cancer Institute (NIH) website. (Table S2.) We create the variable “specialty” according to the cancer site origin. Thoracic surgery included (lung and esophageal cancers), colorectal surgery (colon and rectum cancers), malignant hematology (leukemia), head and neck surgery (head, neck, oral, pharyngeal, and laryngeal cancers), surgical oncology (liver, pancreas, and gastric cancers), urology (kidney and bladder cancers) and, gynecology (cervical cancer). (Table S3). We used respondents with nontobacco‐related cancers as the reference group for all specialties.

### 2.3 Outcomes and procedures

The outcome of interest was the continuation of smoking. This was defined as the proportion of patients currently smokers despite a cancer diagnosis. The exploratory variables were age, gender, education level, income status, marital status, race, and type of specialty for tobacco‐related cancers. BRFSS database is a publicly available database offered by the CDC which contains de‐identified data (http://www.cdc.gov/brfss) and is not categorized as human subjects research, so it does not require an institutional review board approval.

### 2.4 Statistical analysis

Categorical variables were summarized by frequency tables. Chi‐square tests were used to compare two groups of categorical variables. We accounted for complex survey design by adjusting for stratification and clustering at the primary sampling unit. Further, we used sampling weights to calculate the nationwide representative frequencies and proportions. We used multiple imputations via predictive mean matching (*k* = 5 imputations) to address missing values which represented 15.4% for the income variable and less than 0.2% for education, insurance, and gender. We used modified Poisson regression adjusting for age, gender, education, income, race, marital status, and different medical specialties to estimate the relative risk (RR) of being a current tobacco user among cancer survivors who had ever used tobacco. Modified Poison regression was chosen over the more common logistic regression because the incidence of the outcome of interest (continuing smoking) was higher than 10%. Thus, the risk ratio, which is derived from poison regression, is more appropriate than the odds ratio derived from logistic regression.^18^, ^19^ The outcomes of Poisson regression are Risk Ratios to measure the association between the exposures and the outcome accurately. In secondary analyses, we examined interactions with age and education. Sensitivity analyses were performed to evaluate the robustness and consistency of the findings. First, we assessed the impact of considering only tobacco‐related cancers by excluding all nontobacco‐related cancers from the analysis. The comparison group used was malignant hematology, the only nonsurgical specialty among tobacco‐related cancers. Further, we analyzed the different types of cancer by their “origin site” among tobacco‐related cancers. Statistical significance was defined as α <0.05. Data were analyzed using STATA/BE version 17.0.

## 3 RESULTS

### 3.1 Study population

A total of 2,193,981 participants were surveyed between the years 2016 and 2020. We analyzed 19,855 cancer survivors (Weighted: 7,384,612 cancer survivors) who were lifetime or current smokers. As shown in Table 1, active smokers tended to be younger, lower educated, lower income, belong to underrepresented racial minorities, and lack insurance as compared to those participants who quit smoking. Details of the characteristics of the participants are provided in Table 1. There were no statistically significant differences between cohorts. The self‐reported smoking prevalence within the cohort was 26.3%. Among patients with nontobacco‐related cancer, the smoking prevalence was 19.6%. Patients with urological, gynecological, and surgical oncologic cancers related to tobacco use exhibited the highest smoking rate within the population diagnosed with tobacco‐related cancer. (Table 2.)

**TABLE 1:** Comparison of sociodemographic characteristics of lifetime smoking cancer survivors between those who are not currently and currently smoking. (*n* = 19,855).

Cancer survivors characteristics	Not currently smoking (*n* = 14,633)		Currently smoking (*n* = 5222)		chi2	DF
	%	SE	%	SE		
Age					157.9	5
< 40	2.8	0.2	14.8	1.1		
40–49	4.8	0.3	14.8	0.9		
50–59	13.5	0.5	21.7	1.0		
60–69	27.2	0.6	28.8	1.0		
70–79	32.7	0.6	16.8	0.8		
> 80	19.0	0.6	3.1	0.4		
Gender					49.9	1
Female	48.3	0.6	58.5	1.2		
Male	51.7	0.6	41.5	1.2		
Marital Status					136.4	1
Single	36.0	0.7	53.0	1.2		
Married or partner	64.0	0.7	47.0	1.2		
Insurance					230.3	1
Yes	97.5	0.2	89.8	0.8		
No	2.5	0.2	10.2	0.8		
Education level					124.2	2
< High school diploma	10.8	0.5	21.8	1.2		
High school diploma	64.1	0.7	68.1	1.2		
College graduate	25.1	0.5	10.1	0.5		
Race					20.8	3
White	88.7	0.4	82.6	0.9		
Black	5.5	0.3	9.1	0.8		
Hispanic	0.9	0.1	1.9	0.2		
Other	4.9	0.3	6.4	0.6		
Income (USD)					116.2	2
< 25.000	24.0	0.6	45.2	1.2		
25.000–50.000	28.0	0.6	26.5	1.2		
> 50.000	48.0	0.7	28.3	1.1		

*Note*: Estimates reflect weighted data.

**TABLE 2:** Smoking prevalence in cancer survivors by specialty. (*n* = 19,855).

Cancer survivors by specialty type	Distribution		Prevalence	
	%	SE		
			%	SE
Nontobacco‐related cancers	74.8	0.5	19.6	1.2
Tobacco‐related cancers				
Gynecology	6.9	0.4	51.8	0.9
Surgical oncology	1.4	0.1	34.2	0.4
Urology	4.7	0.2	26.2	0.5
Head & neck surgery	1.4	0.1	25.8	0.4
Thoracic surgery	4.6	0.2	25.8	0.6
Colorectal surgery	4.6	0.2	21.8	0.5
Malignant‐hematology	1.2	0.1	19.3	0.2

*Note*: All estimates reflect weighted data.

### 3.2 Predictors of continuing smoking

In our multivariate model, we identify independent predictors for continued smoking in cancer survivors. (Table 3.) The strongest predictor for continued smoking was age which was characterized by younger patients, less than 40 years, having a risk of smoking over nine times higher compared to those older than 80 years ([RR] 9.92; 95% confidence interval [CI]: 7.46–13.9; *p* < 0.001). As compared to college graduates, participants with less than a high school diploma presented twice the risk of smoking (RR 2.06; 95% CI: 1.80 to 2.42; *p* < 0.001). The RR of continuing smoking was 36% higher among single or unmarried participants when compared with those with married or partner status (RR 1.36; 95% CI: 1.26–1.48; *p* < 0.001). Compared with Whites and Hispanic, Black patients were more likely to continue smoking despite the diagnosis of cancer (for Hispanic: RR 1.33; 95% CI: 1.09–1.62; *p* = 0.003; for Black, RR 1.15; 95% CI: 1.01–1.32; *p* = 0.040). Other races and ethnicities did not have a statistically significant association with smoking status following a diagnosis of cancer. There was also no association between smoking status and gender, with similar rates for males and females.

**TABLE 3:** Modified Poisson Multivariate regression‐based association of patient sociodemographic characteristics in continuing to smoke among cancer survivors with a smoking history. (*n* = 19,855).

Factors associated to continue to smoke	Relative risk	95% CI	*p*‐Value
Age			
< 40	9.92	(7.46–13.19)	< 0.001
40–49	9.23	(6.97–12.23)	< 0.001
50–59	7.26	(5.52–9.55)	< 0.001
60–69	5.58	(4.25–7.32)	< 0.001
70–79	3.22	(2.43–4.27)	< 0.001
> 80	(Ref)		
Gender			
Male	1.00	(0.92–1.08)	0.975
Female	(Ref)		
Marital status			
Single	1.36	(1.26–1.48)	< 0.001
Married or partner	(Ref)		
Insurance			
No	1.25	(1.13–1.39)	< 0.001
Yes	(Ref)		
Education level			
< High school diploma	2.09	(1.80–2.42)	< 0.001
High school diploma	1.67	(1.47–1.88)	< 0.001
College graduate	(Ref)		
Race			
White	1.10	(0.96–1.27)	0.142
Black	1.27	(1.06–1.53)	0.009
Hispanic	1.47	(1.17–1.86)	0.001
Other	(Ref)		
Income (USD)			
< 25.000	1.46	(1.31–1.64)	< 0.001
25.000–50.000	1.30	(1.14–1.47)	< 0.001
> 50.000	(Ref)		

*Note*: Estimates reflect weighted data.

### 3.3 Analysis of continuing smoking by specialty

**FIGURE 1:**
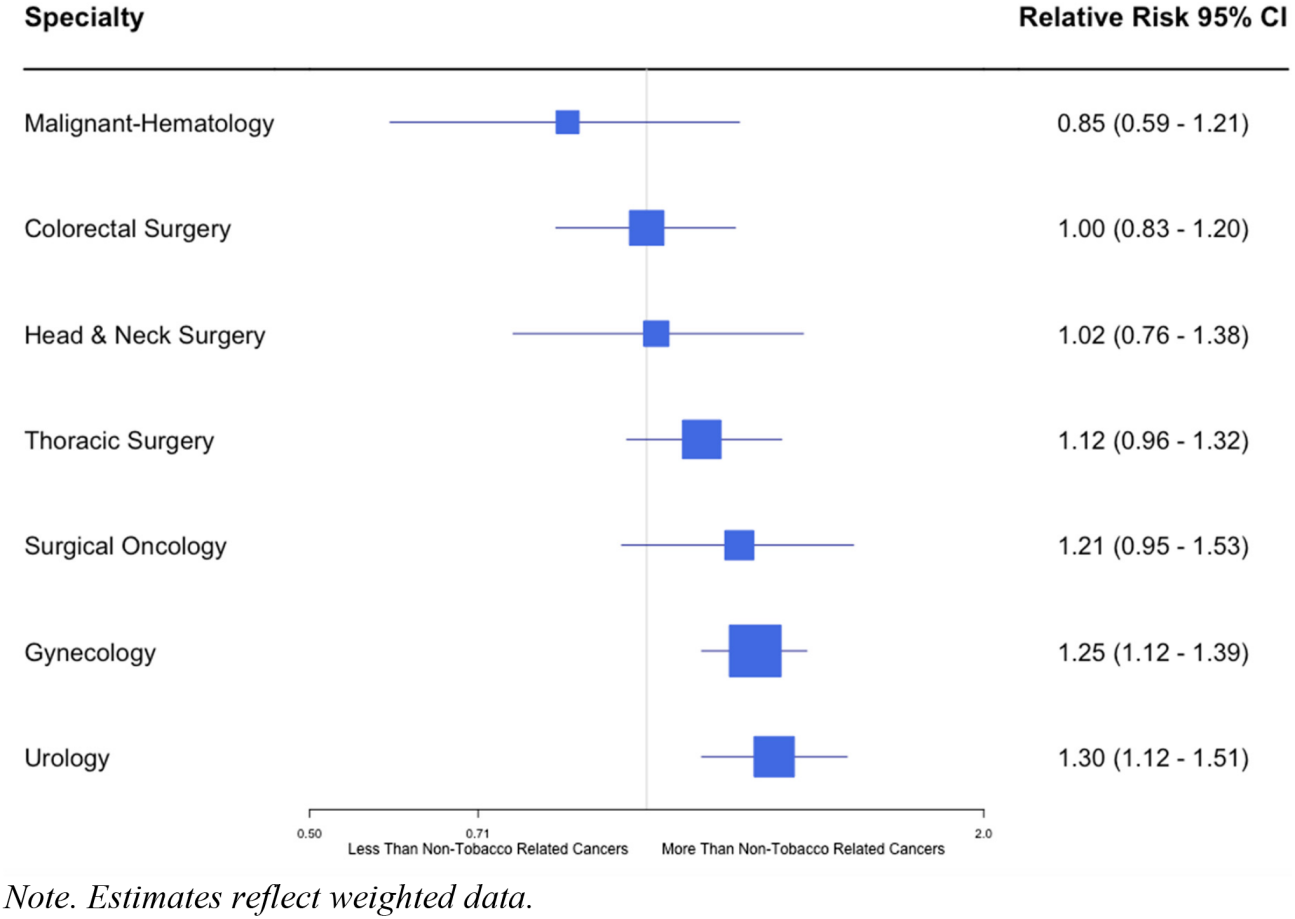
Comparison of the likelihood of continuing to smoke between non‐tobacco‐related cancers and tobacco‐related cancer, by cancer specialty, among lifetime smokers. (*n* = 19,855).

When compared to all nontobacco‐related cancers as a reference group, tobacco‐related cancers have a different tobacco cessation rate by specialty. Malignant Hematology had the lowest RR of smoking continuation among the evaluated specialties (RR 0.85; 95% CI: 0.59–1.21; *p* = 0.370). In contrast, there was a clearly elevated RR for continuing smoking among patients with a diagnosis of a urologic or gynecologic tobacco‐related cancer (RR: 1.30; 95% CI: 1.12–1.51; *p* < 0.001) (RR 1.25; 95% CI: 1.12–1.39; *p* < 0.001) respectively. (Figure 1). Thoracic surgery and surgical oncology tend to have a higher risk of continuing to smoke, although statistical significance was not met to support this association.

### 3.4 Analysis of interactions with age and education

We conducted an interaction analysis by age to investigate whether RRs of continued smoking were consistent between elderly and non‐elderly populations. (Table 4) We found that the overall effect was driven by the non‐elderly population (<65 years) (F7 = 7.5, p_int_ <0.001), with significantly higher RRs observed in urology (RR:1.26, 95% CI 1.27–1.57) and gynecology (RR: 1.41; 1.05–1.52) (F2 = 23.3, *p*_int_ <0.001) and no other significantly elevated RRs among the other TRC specialties (F5 = 0.78, *p*_int_ = 0.567). Furthermore, we found that the overall null finding for thoracic surgery (RR =1.12, 95% CI 0.96–1.32), when disaggregated by elderly and non‐elderly population, was significantly elevated among those over 65 years old (RR = 1.36; 95% CI: 1.07–1.73) and was not elevated in the non‐elderly population. These results suggest that older patients are more likely to continue smoking than their younger counterparts across all cancer specialties studied here, regardless of type, or location within the body.

**TABLE 4:** Comparison of the likelihood of continuing to smoke between nontobacco‐related cancers and tobacco‐related cancer, by cancer specialty, among lifetime smokers: a modified Poisson multivariate regression‐based analysis disaggregated by non‐elderly (<65) and elderly (>65) populations (*n* = 19,855).

	Total distribution		Less than 65 years old			65 years or older		
	%	RR	%	RR	95% CI	%	RR	95% CI
Nontobacco related cancers (Reference)	74.8	1.00	29.5	1.00		45.3	1.00	
Malignant hematology	1.3	0.85	0.7	0.93	(0.63–1.38)	0.6	0. 83	(0.38–1.80)
Colorectal surgery	4.7	1.00	2.2	0.90	(0.73–1.11)	2.5	1.13	(0.79–1.61)
Head & neck surgery	1.4	1.02	0.7	1.13	(0.79–1.62)	0.7	0.96	(0.56–1.65)
Thoracic surgery	4.7	1.12	1.8	0.98	(0.78–1.22)	2.9	1.36	(1.07–1.73)
Surgical oncology	1.5	1.21	0.8	1.22	(0.93–1.61)	0.7	1.65	(0.93–2.85)
		F_5_		0.8			2.0	
		Prob > F_5_ =		0.567			0.078	
Gynecology	6.9	1.25	5.7	1.41	(1.27–1.57)	1.2	1.22	(0.64–2.33)
Urorology	4.7	1.30	1.7	1.26	(1.05–1.52)	3.0	1.19	(0.93–1.53)
		F_2_		23.2			1.1	
		Prob > F_2_ =		< 0.001			0.324	
		F_7_		7.5			1.6	
		Prob > F_7_ =		< 0.001			0.610	

*Note*: Estimates reflect weighted data.

Further, we conducted an interaction analysis by education to investigate whether RRs of continued smoking were consistent between different levels of educational attainment. (Table 5) We found that the overall association between educational attainment and continuing to smoke after diagnosis was driven by the subpopulation with higher levels of education. (High school diploma or more) (F7 = 7.5, *p*_int_ <0.001), with significantly higher RRs observed in urology (RR:1.35, 95% CI 1.15–1.60) and gynecology (RR: 1.36; 1.21–1.53) (F2 = 18.5, *p*_int_ <0.001) and also a significantly elevated RR in surgical oncology (RR:1.49, 95% CI 1.15–1.93) (F5 = 2.23, *p*_int_ = 0.048). These results suggest that individuals with less than a high school diploma were significantly less likely to continue smoking after diagnosis compared to those with higher levels of education.

**TABLE 5:** Comparison of the likelihood of continuing to smoke between non‐tobacco‐related cancers and tobacco‐related cancer, by cancer specialty, among lifetime smokers: a modified Poisson multivariate regression‐based analysis disaggregated by education (< High‐school diploma and High‐school graduate or more) (*n* = 19,855).

	Total distribution		Less than high‐school			High‐school graduate		
	%	RR	%	RR	95% CI	%	RR	95% CI
Non‐tobacco related cancers (Reference)	74.8	1.00	8.6	1.00		66.2	1.00	
Malignant hematology	1.3	0.85	0.2	0.54	(0.25–1.17)	1.1	0.96	(0.66–1.39)
Colorectal surgery	4.7	1.00	0.9	0.77	(0.54–1.10)	3.8	1.11	(0.90–1.38)
Head & neck surgery	1.4	1.02	0.4	0.92	(0.64–1.34)	1.0	1.07	(0.66–1.72)
Thoracic surgery	4.7	1.12	1.0	1.15	(0.89–1.49)	3.7	1.12	(0.93–1.36)
Surgical oncology	1.5	1.21	0.5	0.82	(0.56–1.10)	1.0	1.49	(1.15–1.93)
		F_5_		1.44			2.23	
		Prob > F_5_ =		0.204			0.048	
Gynecology	6.9	1.25	1.4	1.01	(0.83–1.21)	5.5	1.36	(1.21–1.53)
Urorology	4.7	1.30	0.8	1.2	(0.85–1.71)	3.9	1.35	(1.15–1.60)
		F_2_		0.56			18.5	
		Prob > F_2_ =		0.570			< 0.001	
		F_7_		1.23			6.19	
		Prob > F_7_ =		0.284			< 0.001	

*Note*: Estimates reflect weighted data.

### 3.5 Sensitivity analysis

In order to ensure the robustness of our findings and to gain further insight into the smoking status among cancer patients, we conducted several sensitivity analyses. These analyses aimed to assess the impact of different factors and potential confounding variables on our results. By conducting these additional analyses, we could demonstrate the consistency and reliability of our results, even under various scenarios.

First, we sought to evaluate the impact of considering only tobacco‐related cancers on our findings. This was done by excluding all nontobacco‐related cancers from the analysis. The comparison group used in this sensitivity analysis was malignant hematology, as it was the only nonsurgical specialty among the tobacco‐related cancers group. The results of the sensitivity analysis reinforced our original findings as they showed that tobacco‐related cancer survivors with gynecological malignancies had a significantly higher RR (RR: 1.71; 95% CI 1.18–2.46) of continuing to smoke compared to the malignant hematology group. This was also true for patients with urological malignancies, who had a higher RR (RR: 1.48; 95% CI 1.02–2.14) of smoking compared to the malignant hematology group.

Second, we did a sensitivity analysis utilizing the “site of origin” to study the relationship between smoking behavior and different types of cancer. The grouping of tobacco‐related cancers based on their site of origin allowed for a more in‐depth examination of potential differences in smoking behavior across various cancer types and the identification of confounding factors. Isolating the impact of each cancer site type enhanced our understanding of the connection between smoking and cancer, thereby reinforcing the robustness of the original findings. The results of this sensitivity analysis showed that patients with cervical cancer (RR: 1.70; 95% CI 1.18–2.46) and urological cancers (Bladder and Kidney) (RR: 1.48; 95% CI 1.02–2.15) had a significantly higher RR of continuing to smoke compared to the malignant hematology group. These findings further support our original findings and underscore the need for increased attention to smoking cessation interventions for patients with these cancer types.

## 4 DISCUSSION

This study found that more than one‐fourth (26.3%) of cancer survivors were current smokers. While our results are consistent with previously reported findings between younger age and lower education associated with smoking in tobacco‐related cancer patients,^12^, ^13^, ^16^, ^20^, ^21^ we interestingly found that the rate of individuals continuing to smoke varied according to the specialty involved in cancer care thus highlighting the likely important role of the healthcare professional with respect to tobacco cessation. It is feasible that these findings are due to a difference in tobacco cessation counseling by the treating physician.^22^ Specifically, we identified that having urological or gynecological cancer was significantly associated with continuation of smoking after a cancer diagnosis. These findings suggested that urologists and gynecologists might not offer adequate counseling about tobacco cessation to patients compared to other specialists.

The strengths of this study include the analysis of the world's largest continuously conducted health survey system, including five recent years. Thus, our findings largely reflect the current status regarding smoking attitudes among cancer patients in the United States. To the best of our knowledge, our study is the first nationwide study to identify differential rates of tobacco use among patients with tobacco‐related cancers. However, our results are supported by a recent national sample study where 1058 physicians from six different specialties were surveyed regarding adherence to the U.S Public Health Service (USPHS) Clinical Practice tobacco cessation guidelines, Schaer et al. found overall low adherence (18%) as well as differential implementation among the medical specialties.^23^

Our study identified urology and gynecology as the specialties with the highest rates of patients continuing to smoke despite their cancer diagnosis. According to a national survey centered on urologists in the United States, only 19.8% of respondents declared always discussing tobacco cessation with their bladder cancer patients.^24^ This low rate of providing smoking cessation assistance may explain the higher rate of continuing smoking we found in urologic cancer survivors. Additionally, Swoboda et al. analyzed the Health Information National Trends Survey and reported a continuing smoking prevalence of 31.2% in cervical cancer survivors, representing the highest prevalence of all cancers.^13^ This also supports our findings about patients with a gynecologic malignancy having a significantly higher probability of continuing to smoke despite the diagnosis of a tobacco‐related cancer. It is plausible that this could be related to the fact that gynecologists deal with cervical cancer, which represents a cluster of patients with higher‐risk health behavior, and it might be harder to counsel this particular group against tobacco use because of their unfavorable socioeconomic and educational status.^25^, ^26^ Another possible explanation might be the perception of disease both by the patient and the specialist. Both urology and gynecology specialties have an important proportion of endoscopic outpatient procedures compared to other specialties. These short endoscopic procedures, such as transurethral resection of the bladder or cervical conization, are usually low‐complexity procedures, which do not leave any visible scars, and patients have a speedy recovery, often being discharged on the same day to their homes. This fast treatment and the shorter interaction with the physician could, in part, create fewer opportunities for counseling and explain the difference in the smoking rates in the survivors of these specialties.

We found that malignant hematology patients had the highest rate of tobacco cessation among the specialties in the current study. Importantly, out of all studied specialties in this study, hematologists were the only ones who had an internal medicine background compared to all other surgical specialties. Shaer et al. found that internists had the highest rates of implementation of arranging for follow‐up for tobacco cessation.^23^ Another possible reason could be that leukemia treatment usually lasts several months, and patients are admitted to hospitals for long periods creating more opportunities to discuss tobacco cessation with patients.

The importance of tobacco cessation in cancer survival has been widely studied and associated with a positive impact to a great extent. Some studies show that quitting smoking is associated with better tolerance of treatments and improvement in treatment outcomes,^27^, ^28^, ^29^, ^30^, ^31^, ^32^ as well as fewer treatment complications.^33^ Additionally, there is robust evidence that quitting smoking is associated with a substantial reduction in the risk of recurrence and progression.^4^, ^5^, ^6^, ^34^, ^35^ Foerster et al. examined in a systematic review and meta‐analysis the association between smoking status and the risk of biochemical recurrence, metastasis, and cancer‐specific mortality among patients with localized prostate cancer undergoing treatment. Within the median follow‐up of 72 months, current smokers had a 40% higher risk of biochemical recurrence, a significantly higher risk of metastasis (HR, 2.51; 95% CI, 1.80–3.51), and an 89% higher risk of cancer‐specific mortality^36^ Furthermore, smoking cessation reduces the risk of a second primary malignancy,^37^, ^38^, ^39^ and is associated with better quality of life and longer survival.^7^, ^8^, ^40^, ^41^, ^42^ This highlights the importance of providing extra support to those high‐risk cancer populations.

Our results have implications for clinical practice, as they identify the characteristics of those patients who might require a special tobacco‐cessation approach during their follow‐up. Clinicians must obtain the necessary training to provide comprehensive tobacco cessation counseling to every patient and more intense counseling to cancer survivors and encourage them to engage in a tobacco cessation program.^43^ It was established that the best time to encourage a patient to quit smoking is at the time of the cancer diagnosis; this is known as “the teachable moment,” opportunity healthcare professionals should take advantage of.^44^, ^45^, ^46^ A multidisciplinary approach to these patients must be offered to grasp this unique opportunity. The difference in tobacco cessation observed among specialties could serve as a call to action for societies and physicians to reflect on current tobacco cessation approaches offered to their patients.

Our paper has some limitations. First, this was a cross‐sectional study, and we did not consider temporality; therefore, causality cannot be established. We do not know if some patients quit smoking before the cancer diagnosis. Nevertheless, this may not be a major drawback when investigating differences in smoking prevalence between patients that have ever been in contact with a physician of a particular specialty. Second, the BRFSS database is constructed by self‐reported retrospective information that might be affected by recall and nonresponse bias. Nonetheless, the “guilt and shame” of patients who had tobacco‐related cancer and continue to smoke might affect their report rates, underestimating the real prevalence of tobacco use in this population.^47^ Third, we do not measure the number of packs/years, so we cannot determine whether participants were light or heavy smokers. However, for both questions regarding previous and current smoking status, the participant had the possibility to respond, “Don't Know/Not Sure.” Fourth, even though we were able to do an analysis comparing different specialties, we could not examine differences among physicians' characteristics. We believe certain factors, such as time since graduating from medical school, working in an academic hospital, and treating high‐volume tobacco‐related cancer patients, among others, might be associated with different smoking cessation counseling rates. Further research focusing on physician education and practice on smoking cessation is warranted. Fifth, our study may have been affected by right‐censoring due to the potential underrepresentation of severe cancer cases related to certain specialties, which could be linked to higher smoking rates and quantity. Nevertheless, this is data from the most extensive national survey available, providing a comprehensive overview of cancer survivors' current situation that can be used to identify key areas for improvement and opportunities for tobacco cessation counseling.

In conclusion, this comprehensive examination found that one in four cancer survivors were current smokers and demonstrated a different rate of continued smoking among cancer survivors by specialties. These findings may aid societies in focusing on tobacco cessation counseling in guidelines and recommendations. It will also help physicians identify patients with a higher risk of continuing smoking after cancer diagnosis and who might need personalized tobacco cessation counseling.

## AUTHOR CONTRIBUTIONS

**José Ignacio Nolazco:** Conceptualization (lead); data curation (lead); formal analysis (lead); investigation (lead); methodology (equal); project administration (equal); visualization (equal); writing – original draft (lead); writing – review and editing (equal). **Yuzhe Tang:** Conceptualization (supporting); data curation (equal); formal analysis (equal); investigation (equal); methodology (supporting); writing – review and editing (equal). **Khalid Y Alkhatib:** Data curation (supporting); investigation (supporting); resources (equal); software (equal); writing – review and editing (equal). **Andrew J King:** Data curation (supporting); formal analysis (supporting); methodology (supporting); visualization (supporting); writing – review and editing (equal). **Matthew Mossanen:** Conceptualization (equal); investigation (equal); supervision (equal); validation (lead); visualization (equal); writing – review and editing (equal). **Steve Chang:** Conceptualization (equal); data curation (supporting); formal analysis (equal); investigation (equal); methodology (equal); project administration (equal); resources (lead); software (equal); supervision (lead); validation (supporting); visualization (supporting); writing – original draft (supporting); writing – review and editing (lead).

## FUNDING INFORMATION

This research was conducted with no funding.

## CONFLICT OF INTEREST STATEMENT

None.

## Supporting information


**Table S1.** Smoking Status Assessment
**Table S2.** Tobacco‐Related Cancers, from National Cancer Institute (NIH)
**Table S3.** Medical Specialty Variable

## Data Availability

Data derived from public domain resources. The data that support the findings of this study are available in CDC webpage at https://www.cdc.gov/brfss/index.html. These data were derived from the following resources available in the public domain: https://www.cdc.gov/brfss/annual_data/annual_data.htm
