# Correction to “Smoking status among cancer patients by medical specialty: A U.S. nationwide representative analysis”

**DOI:** 10.1002/cam4.7299

**Published:** 2024-05-20

Nolazco JI, Tang Y, Alkhatib KY, King AJ, Mossanen M, Chang SL. Smoking status among cancer patients by medical specialty: A U.S. nationwide representative analysis. Cancer Med. 2023; 12: 21389‐21399. doi:10.1002/cam4.6684

The article title has been corrected to read: “Smoking status among cancer patients by medical specialty: A U.S. nationwide representative analysis.”

The word “medical” has been added before “specialty.”

We apologize for this error.

