# Supplementary material for: Smoking status among cancer patients by specialty: A U.S. nationwide representative analysis
Source: Cancer Med. 2023 Nov 20;12(23):21389–99. doi: 10.1002/cam4.6684 (PMC10726820; doi:10.1002/cam4.6684)
Supplement: Supplementary file 1 — Table S1. Smoking Status Assessment Table S2. Tobacco‐Related Cancers, from National Cancer Institute (NIH) Table S3. Medical Specialty Variable [file CAM4-12-21389-s001.docx]

**Supplementary Material**

**Table S1.**

*Smoking Status Assessment*

| SMQ100 Have you ever smoked more than 100 cigarettes in your life? | | | |
| --- | --- | --- | --- |
| 1 | Yes | Smoking History | |
| 2 | No | Never Smokers | |
| SMOKDAY2 Do you now smoke cigarettes every day, some days, or not at all? | | | |
| 1 | Every day | Current Smokers | |
| 2 | Some days | Current Smokers | |
| 3 | Not at all | Past Smokers | |

*Note. Patients who report being “Never Smokers” were excluded from the analysis. Patients who had smoked at least 100 cigarettes in their lives were considered “Ever Smokers.” Those who ever smoked were further divided into current and former smokers according to the question SMOKDAY2.*

**Table S2.**

*Tobacco-Related Cancers, from National Cancer Institute (NIH)*

| **Tobacco-Related Cancers** |
| --- |
| Lung |
| Larynx (voice box) |
| Mouth |
| Esophagus |
| Throat |
| Bladder |
| Kidney |
| Liver |
| Stomach |
| Pancreas |
| Colon |
| Rectum |
| Cervix |
| Acute Myeloid Leukemia |

*Note.* Adapted from the Nation Cancer Institute (NIH) website. <https://www.cancer.gov/about-cancer/causes-prevention/risk/tobacco#:~:text=Tobacco%20use%20causes%20many%20types,well%20as%20acute%20myeloid%20leukemia>

**Table S3.**

Medical Specialty Variable

| **Specialty** | **Cancer Site** |
| --- | --- |
| Colorectal Surgery | Colon, and Rectum |
| Gynecology | Cervical |
| Head & Neck Surgery | Oral, Pharyngeal, and Larynx |
| Malignant Hematology | Leukemia |
| Surgical Oncology | Liver, Pancreas, and Stomach |
| Thoracic Surgery | Lung, and Esophageal |
| Urology | Kidney, and Bladder |

*Note.* Medical Specialties according to the site of the Tobacco-Related cancer
